# Non-suicidal self-injury and subsequent suicide attempts and death: A protocol for a systematic review of temporally ordered evidence

**DOI:** 10.1371/journal.pone.0357977

**Published:** 2026-09-10

**Authors:** Anvar Sadath, Muhammed Thalil, Susan Reay, Jaiden Davis, Zubair Kabir

**Affiliations:** 1 Grace Abbott School of Social Work, College of Public Affairs and Community Service, University of Nebraska at Omaha, Omaha, Nebraska, United States of America; 2 Population Research Institute, Center for Health Ageing, The Pennsylvania State University, University Park, Pennsylvania, United States of America; 3 School of Public Health, University College Cork, Cork, Ireland; South China Normal University, CHINA

## Abstract

**Background:**

Non-suicidal self-injury (NSSI) is a prevalent and clinically significant behaviour, particularly among adolescents and young adults, and is strongly associated with suicidal behaviour. However, existing reviews have often combined heterogeneous outcomes, including suicidal ideation, plans, attempts, and death, limiting the identification of clinically meaningful pathways.

**Objective:**

This protocol describes a systematic review that aims to synthesise evidence on the temporally ordered relationship between NSSI and subsequent suicide attempts or death, and to examine whether characteristics of NSSI are associated with increased risk or severity of later suicidal behaviour.

**Methods:**

This protocol is reported in accordance with PRISMA-P 2015 and registered with the Open Science Framework (OSF; https://doi.org/10.17605/OSF.IO/EA4M6). A comprehensive search will be conducted in MEDLINE, Embase, PsycINFO, CINAHL, and Web of Science. Eligible studies will include prospective and retrospective observational studies demonstrating temporal ordering between NSSI and subsequent suicide attempts or death. Studies focusing solely on suicidal ideation will be excluded. Data extraction and risk-of-bias assessment will be conducted independently by two reviewers using Joanna Briggs Institute tools. Due to anticipated heterogeneity, findings will be synthesised using a structured narrative approach.

**Conclusion:**

This review will provide a focused synthesis of evidence on the relationship between NSSI and subsequent suicidal outcomes, addressing gaps related to temporality, outcome specificity, and severity. The findings are expected to inform clinical formulation, safety planning, and future research on suicide prevention by improving understanding of factors associated with subsequent suicidal behaviour.

## Background

Self-injurious behaviours represent a major public health concern, particularly among adolescents and young adults [1]. Among these, non-suicidal self-injury (NSSI), defined as the deliberate, direct, and self-inflicted destruction of body tissue without suicidal intent and for purposes not socially sanctioned (e.g., body piercing, tattooing), has emerged as a distinct clinical and research construct [2,3]. Common methods of NSSI include cutting, hitting, scraping, burning, stabbing, and biting – with cutting (using knives) being the most prevalent practice [4], although emerging evidence indicates increased prevalence of scratching particularly in the clinical samples of adolescent and young adults [5].

NSSI is widely recognised as distinct from suicide attempts in terms of intent, lethality, function, and clinical presentation [6,7]. In general, the most important distinction is the intention [8]. While suicide attempts are characterized by an intent to die, NSSI is typically performed to regulate emotional distress, reduce psychological tension, or cope with overwhelming affective states [8]. Despite these differences, a substantial body of research demonstrates that NSSI and suicidal behaviours are closely associated and frequently co-occur across both clinical and community populations [9]. Estimates suggest that a large proportion of individuals with a history of NSSI also report suicide attempts, highlighting the clinical importance of understanding this relationship [9].

Importantly, emerging evidence suggests that NSSI may not only co-occur with suicidal behaviour but may also precede and predict it [10]. Longitudinal and retrospective findings indicate that NSSI often occurs earlier in development than suicide attempts, with an observable temporal gap between onset of NSSI and subsequent suicidal behaviour [9,10]. This temporal sequencing has led to increasing interest in NSSI as a potential precursor to subsequent suicidal behaviour [9]. The present review specifically focuses on the temporal transition from NSSI to subsequent suicide attempts or death, with particular emphasis on whether NSSI predicts not only occurrence but also greater lethality or clinical severity of later suicidal behaviour

Theoretical models provide an important framework for understanding the progression from NSSI to more severe suicidal behaviour. The Interpersonal Theory of Suicide proposes that repeated exposure to painful and provocative experiences, such as NSSI, may increase an individual’s acquired capability for suicide through habituation to physical pain and reduced fear of death [11,12]. Consistent with this theory, characteristics of NSSI, including greater frequency, repetition, and engagement in multiple methods, have been associated with later suicide attempts and more severe suicidal outcomes in some studies [13,14].

The Integrated Motivational–Volitional (IMV) Model complements this perspective by distinguishing factors involved in the emergence of suicidal ideation from those that facilitate the transition to suicidal behaviour [15]. Together, these theoretical models provide a rationale for examining the temporal relationship between NSSI and subsequent suicide attempts or death. However, the extent to which longitudinal and retrospective evidence supports this relationship has not been comprehensively synthesised.

Although prior reviews have established an association between NSSI and suicidal behaviour, important limitations remain. Narrative syntheses have primarily focused on shared risk factors and conceptual relationships rather than temporally ordered pathways [16]. Meta-analytic evidence from longitudinal studies has examined self-injurious thoughts and behaviours as a broad category, often combining suicidal ideation, plans, attempts, and death into a single outcome, thereby limiting the ability to isolate NSSI-specific trajectories or clinically meaningful temporal relationship [17]. Other reviews have focused on co-occurrence [18] or the longitudinal course of NSSI itself rather than its role as a predictor of subsequent suicidal outcomes [3].

While some previous reviews have examined specific aspects of the relationship between NSSI and suicidal behaviour, these have generally been limited in scope, included relatively small numbers of studies, or focused on selected outcomes rather than providing a comprehensive synthesis of temporally ordered evidence [7]. In addition, previous reviews have not systematically examined important dimensions such as suicide death, method lethality, or clinical severity. Furthermore, recent work highlights the limited availability of prospective evidence and the need for a better understanding of the temporal relationship between NSSI and subsequent suicidal behaviour [9].

Severity of suicide attempts may be reflected in indicators such as method lethality, degree of medical injury, or the need for clinical intervention, each representing distinct dimensions of risk. Importantly, combining heterogeneous outcomes -particularly suicidal ideation with suicide attempts and death—may obscure clinically meaningful differences in risk processes and severity. In contrast, suicide attempts and suicide death are behaviourally enacted outcomes with direct clinical and public health relevance.

To our knowledge, no previous systematic review has comprehensively synthesised observational evidence while simultaneously: (1) defining NSSI as an exposure of interest (2) restricting outcomes to temporally ordered to suicide attempts or death, and (3) examining indicators of method lethality or clinical severity.

By integrating these elements within a single systematic review, the present study is expected to provide a more focused understanding of the temporal relationship between NSSI and subsequent suicidal outcomes and to strengthen the evidence base for clinical formulation, safety planning, and future suicide prevention research. Contemporary clinical guidance recommends moving beyond categorical suicide risk stratification towards comprehensive psychosocial assessment, clinical formulation, and safety planning [19,20].

### Primary review question

Among individuals with a history of non-suicidal self-injury, what is the evidence for a temporal relationship between NSSI and subsequent suicide attempts or death based on prospective or retrospective studies?

### Secondary review questions

Within studies demonstrating temporal ordering:

To what extent do characteristics of NSSI (e.g., frequency, repetition, number of methods, or recency) influence the risk of subsequent suicide attempts or death?
Is prior NSSI associated with escalation in the lethality or clinical severity of subsequent suicide attempts (e.g., method lethality, medical consequences, or need for clinical intervention)?

## Methods

This protocol describes a systematic review examining the temporal relationship between non-suicidal self-injury (NSSI) and subsequent suicide attempts or death, with particular attention to indicators of lethality and clinical severity. The protocol has been developed in accordance with the Preferred Reporting Items for Systematic Review and Meta-Analysis Protocols (PRISMA-P) 2015 guidelines (S1 Checklist). The completed review will be reported following the PRISMA 2020 statement.

This systematic review protocol was prospectively registered with the Open Science Framework (OSF) Registries (https://doi.org/10.17605/OSF.IO/EA4M6). Any amendments to the protocol will be documented with the date, description of the change, and rationale, and updated in the registry.

### Eligibility criteria

Eligibility criteria are structured using a Population–Exposure–Comparator–Outcome–Study design (PECO) framework.

### Population

Studies will be eligible if they include individuals of any age who have engaged in self-injurious behaviours explicitly reported without suicidal intent. Eligible populations may include clinical samples, community samples, school-based populations, emergency department cohorts, and population-based datasets.

Studies focusing exclusively on suicidal ideation/thinking or planning without behavioural self-injury will be excluded.

### Exposure

The exposure of interest is non-suicidal self-injury (NSSI), defined as deliberate, self-inflicted injury without suicidal intent, as specified by the original study authors.

Eligible exposures include:

- NSSI assessed at baseline in prospective studies
- history of NSSI (e.g., lifetime or past-year) in retrospective studies
- self-injurious behaviour where suicidal intent is explicitly assessed and reported as absent

Studies using broader terms such as “self-harm” or “deliberate self-harm” will be included only when suicidal intent is explicitly assessed and reported as absent, or when data relating specifically to non-suicidal self-injury can be clearly identified and extracted. Studies in which suicidal intent cannot be determined will be excluded to minimise misclassification. Studies will not be required to use DSM-5 diagnostic criteria for NSSI because the review aims to include the full body of observational evidence, including studies published before DSM-5. Instead, eligibility will be based on the defining feature of NSSI—the absence of suicidal intent.

### Comparator

Where available, comparators may include:

- individuals without a history of NSSI
- individuals with varying frequency, repetition, or severity of NSSI
- within-person comparisons across time

Studies without formal comparator groups will be included if they report associations between NSSI and subsequent outcomes.

### Outcomes

#### Primary outcomes.

- Subsequent suicide attempts
- Death by suicide

Suicide attempts will be defined according to the original study authors as self-injurious or self-harm behaviour with at least some intent to die. Death by suicide will be defined as fatal self-inflicted injury with evidence of intent.

Studies reporting only suicidal ideation/thinking or planning without behavioural outcomes will be excluded.

#### Secondary outcomes.

Indicators of severity or lethality in subsequent suicide attempts, including:

- use of higher-lethality methods (e.g., hanging, firearms, jumping)
- medically serious attempts (e.g., requiring emergency or surgical intervention)
- hospitalization or intensive care unit (ICU) admission
- clinician-rated or scale-based lethality or severity

Where definitions vary across studies, operational definitions will be extracted and categorised to reflect distinct dimensions of severity.

### Study design and report characteristics

Eligible study designs include:

- prospective (longitudinal) cohort studies
- retrospective cohort or case–control studies

All included studies must demonstrate that NSSI occurred before the subsequent suicide attempt or death. Temporal ordering will be considered established when the study clearly reports that NSSI preceded the suicidal outcome through prospective follow-up or retrospective evidence, such as documented age of onset, event history, medical records, or other clearly reported chronological information. Although retrospective studies relying on self-reported histories may be subject to recall bias, they will be considered eligible when the temporal sequence is explicitly described by the original study authors. Potential limitations related to recall bias and the lower inferential strength of retrospective designs will be considered during risk-of-bias assessment and interpretation of the findings. Cross-sectional studies will be excluded due to their limited ability to establish temporal ordering and the risk of recall bias. Restricting inclusion to longitudinal and retrospective designs is expected to strengthen the validity and interpretability of findings regarding the temporal relationship between NSSI and subsequent suicide attempts or death, although it may reduce the number of eligible studies. No minimum time interval between NSSI and the subsequent suicidal outcome will be specified as an eligibility criterion. This decision reflects the variability in follow-up periods across observational studies and the absence of an established threshold defining the time interval between NSSI and subsequent suicidal behaviour. Applying an arbitrary cut-off could exclude otherwise relevant studies and reduce the completeness of the evidence synthesis. Instead, the reported interval between NSSI and the subsequent suicide attempt or death will be extracted whenever available and considered when interpreting the findings.

Qualitative-only studies, case reports, case series, editorials, commentaries, dissertations, conference abstracts, and intervention trials without relevant observational data will be excluded. Only peer-reviewed journal articles will be included.

No restrictions will be applied based on year of publication. No language restrictions will be applied. Where necessary, non-English articles will be translated using appropriate online translation tools and, where feasible, verified by individuals with proficiency in the relevant language. If a study cannot be adequately translated for eligibility assessment or data extraction, the reason for exclusion will be documented and reported in the review.

### Information sources

The following electronic databases will be searched from inception to the date of the final search:

- MEDLINE (via PubMed)
- Embase
- PsycINFO
- CINAHL
- Web of Science Core Collection

Reference lists of included studies and relevant reviews will also be screened to identify additional eligible studies.

The literature search will be updated immediately before completion of the review to identify any newly published eligible studies.

### Search strategy

The search strategy will combine controlled vocabulary (e.g., MeSH, Emtree) and free-text terms related to non-suicidal self-injury (NSSI), suicide attempts and death, and longitudinal or temporally informative study designs.

A model search strategy developed for PubMed is provided in S2 file. This strategy was constructed by combining three concept blocks: (1) non-suicidal self-injury, (2) suicide attempts and death, and (3) longitudinal or temporally informative study designs. The PubMed strategy will be adapted appropriately for use in other databases.

No date limits will be applied. Full database-specific search strategies will be reported in the final review to ensure transparency and reproducibility.

### Data management

Search results will be exported and imported into Rayyan for deduplication and screening. Automated duplicate detection will be used, followed by manual verification. Screening decisions will be documented with audit trails.

### Selection process

Titles and abstracts will be screened independently by two reviewers against the eligibility criteria. Full texts of potentially eligible studies will be assessed independently by the same reviewers.

Disagreements will be resolved through discussion, with consultation of a third reviewer if needed. The selection process will be documented using a PRISMA flow diagram.

### Data collection process

A standardized data extraction form will be developed and pilot-tested. Data extraction will be conducted independently by two reviewers. Discrepancies will be resolved by consensus.

Where necessary, study authors may be contacted for clarification or missing data.

### Data items

- Study characteristics (author, year, country, design)
- Sample characteristics (age, sex, population type)
- Definitions and measurement of NSSI, terminology used to describe suicidal behaviours, operational definitions of suicide attempts and suicide death, and the methods used by study authors to determine suicidal intent.
- Method of assessing suicidal intent
- Exposure classification (baseline NSSI vs history of NSSI)
- Outcome definitions (suicide attempts, death, severity indicators)
- Follow-up duration, the reported interval between NSSI and the suicidal outcome (where available), and the method used to establish temporal ordering. Effect estimates (e.g., odds ratios, hazard ratios) with corresponding confidence intervals and p-values (where available)
- Covariates included in adjusted models
- Risk-of-bias indicators (based on Joanna Briggs Institute tools)

Where multiple models are reported, the most fully adjusted estimates will be extracted.

Where multiple publications from the same cohort are identified, the studies will be examined to determine the extent of sample overlap. Where substantial overlap exists, data will be extracted only once for each outcome to avoid double counting. Preference will be given to the publication with the largest sample size, longest follow-up, or most comprehensive reporting of the outcome of interest. Additional publications from the same cohort may be used to supplement methodological or outcome information where relevant.

### Risk of bias assessment

Methodological quality will be assessed independently by two reviewers using the Joanna Briggs Institute critical appraisal tools [21] appropriate for study design, including tools for cohort and case–control studies. The JBI critical appraisal tools were selected because they provide validated design-specific instruments for assessing the methodological quality of observational studies, allowing a consistent approach across the prospective cohort, retrospective cohort, and case-control studies included in this review [22,23].

Disagreements will be resolved through discussion or consultation with a third reviewer. Risk-of-bias assessments will inform interpretation but will not be used as exclusion criteria.

### Assessment of certainty of evidence

The overall certainty of the evidence will be assessed using the Grading of Recommendations Assessment, Development and Evaluation (GRADE) approach, adapted, where appropriate, for observational prognostic evidence. The assessment will consider the domains of risk of bias, inconsistency, indirectness, imprecision, and publication bias. Given the anticipated heterogeneity in study designs, exposure definitions, and outcome measures, the certainty assessment will be used to support the interpretation of the overall body of evidence rather than to inform clinical recommendations.

### Data synthesis

A structured narrative synthesis will be undertaken because substantial heterogeneity is anticipated in study designs, definitions and measurement of NSSI, assessment of suicidal intent, outcome definitions, follow-up duration, and reported effect measures. These differences are expected to limit the comparability of studies and the appropriateness of quantitative pooling. If, during the review process, a sufficient number of studies are identified that are methodologically comparable with respect to study design, exposure definition, outcome measurement, and effect estimates, the feasibility of a quantitative synthesis will be considered. The decision to undertake a meta-analysis will therefore be based on the degree of clinical and methodological homogeneity observed among the included studies.

Studies will be grouped and synthesised according to:

- study design (prospective and retrospective with clearly defined temporal sequencing)
- exposure classification (baseline-defined NSSI vs history of NSSI)
- outcome type (suicide attempts, suicide death, and indicators of lethality or clinical severity)

Given the diversity of outcomes reported across studies, suicide attempts, suicide death, and indicators of lethality or clinical severity will be interpreted as distinct outcome constructs during the narrative synthesis. Where operational definitions vary across studies, these differences will be considered when interpreting the findings and will not be treated as interchangeable measures of suicidal behaviour.

The synthesis will be structured to address the primary and secondary review questions:

1. evidence on the temporal relationship between NSSI and subsequent suicide attempts or death
2. influence of NSSI characteristics (e.g., frequency, repetition, number of methods, recency) on subsequent outcomes
3. evidence of escalation in lethality or clinical severity

Findings will be compared across studies to assess:

- consistency of associations
- direction and magnitude of effects (where reported)
- influence of study design and measurement approaches

Particular attention will be given to:

- how NSSI is defined and operationalised
- how suicidal intent is assessed
- how temporal relationships are established
- how severity and lethality are measured

Given the recognised variation in terminology used to describe suicidal behaviours, the operational definitions and nomenclature adopted by individual studies will also be compared during data synthesis. Interpretation of findings will be informed by international recommendations on the nomenclature of suicidal behaviours by De Leo et al [24], while preserving the original definitions reported in the included studies. This approach will facilitate transparent interpretation of findings without reclassifying outcomes reported by the original authors.

The strength of evidence will be interpreted in relation to study design, with greater weight given to prospective studies due to their stronger temporal structure.

## Discussion

Non-suicidal self-injury (NSSI) represents a significant public health concern, particularly among adolescents and young adults, and is strongly associated with subsequent suicidal behaviour [1,17]. However, existing research has largely focused on broad and heterogeneous outcomes, often combining suicidal ideation, attempts, and death, which limits the ability to identify clinically meaningful temporal relationships. By focusing specifically on behaviourally enacted outcomes—suicide attempts and death—and requiring clearly established temporal ordering based on longitudinal and retrospective designs, this review aims to provide a more precise understanding of the temporal relationship between NSSI and subsequent suicide attempts or death.

The findings of this review are expected to have implications for clinical practice, research, and policy. By clarifying the temporal relationship between NSSI and subsequent suicide attempts or death, this review may contribute to evidence supporting comprehensive psychosocial assessment, clinical formulation, and safety planning, consistent with contemporary clinical guidance [19,20], rather than reliance on categorical suicide risk stratification alone. From a research perspective, the review will identify methodological limitations in the existing literature, including inconsistencies in the definition and measurement of NSSI and the limited availability of longitudinal evidence [7,16]. At a policy level, the findings may help inform future suicide prevention strategies by highlighting the importance of distinguishing between suicidal ideation and behaviour and by identifying areas where further prospective research is needed.

Although the review will rely on observational evidence, specifically prospective and retrospective study designs, and will employ a narrative synthesis approach, which may limit causal inference, the use of stringent inclusion criteria is intended to enhance conceptual clarity and internal validity. By restricting inclusion to studies that demonstrate temporal ordering between NSSI and subsequent suicidal outcomes, this review aims to provide a more robust assessment of temporal relationship. These criteria may reduce the number of eligible studies; however, they are expected to strengthen the interpretability and validity of findings on subsequent suicide attempts and death following NSSI. Restricting the review to peer-reviewed journal articles may increase the risk of publication bias by excluding unpublished studies, dissertations, and conference abstracts, particularly if studies reporting null findings are less likely to be published. This limitation will be considered when interpreting the review findings.

## Supporting information

S1 Checklist PRISMA-P 2015 checklist. (DOCX)

S2 File PubMed search strategy. (DOCX)
