# Supplementary material for: Non-suicidal self-injury and subsequent suicide attempts and death: A protocol for a systematic review of temporally ordered evidence
Source: PLoS One. 2026 Sep 10;21(9):e0357977. doi: 10.1371/journal.pone.0357977 (PMC13561375; doi:10.1371/journal.pone.0357977)
Supplement: Supplementary file 2 [file pone.0357977.s002.docx]

**S2 File. PubMed search strategy**

The PubMed search strategy combined controlled vocabulary (MeSH) and free-text terms related to non-suicidal self-injury, suicide attempts and death, and longitudinal study designs.

**Search 1 (NSSI terms):**

"Self-Injurious Behavior"[Mesh]
OR "non-suicidal self injur*"[Title/Abstract]
OR "nonsuicidal self injur*"[Title/Abstract]
OR NSSI[Title/Abstract]
OR "self-harm"[Title/Abstract]
OR "self harm"[Title/Abstract]
OR "deliberate self-harm"[Title/Abstract]
OR "deliberate self harm"[Title/Abstract]

**Search 2 (Suicide outcomes):**

"Suicide, Attempted"[Mesh]
OR "suicide attempt*"[Title/Abstract]
OR "attempted suicide"[Title/Abstract]
OR "completed suicide"[Title/Abstract]
OR "suicide death"[Title/Abstract]

**Search 3 (Study design):**

"Longitudinal Studies"[Mesh]
OR "Prospective Studies"[Mesh]
OR "Cohort Studies"[Mesh]
OR longitudinal[Title/Abstract]
OR prospective[Title/Abstract]
OR cohort[Title/Abstract]
OR "follow-up"[Title/Abstract]
OR "follow up"[Title/Abstract]

**Final combined search:**

Search 1 AND Search 2 AND Search 3

("Self-Injurious Behavior"[Mesh] OR "non-suicidal self injur*"[Title/Abstract] OR "nonsuicidal self injur*"[Title/Abstract] OR NSSI[Title/Abstract] OR "self-harm"[Title/Abstract] OR "self harm"[Title/Abstract] OR "deliberate self-harm"[Title/Abstract] OR "deliberate self harm"[Title/Abstract])
AND
("Suicide, Attempted"[Mesh] OR "suicide attempt*"[Title/Abstract] OR "attempted suicide"[Title/Abstract] OR "completed suicide"[Title/Abstract] OR "suicide death"[Title/Abstract])
AND
("Longitudinal Studies"[Mesh] OR "Prospective Studies"[Mesh] OR "Cohort Studies"[Mesh] OR longitudinal[Title/Abstract] OR prospective[Title/Abstract] OR cohort[Title/Abstract] OR "follow-up"[Title/Abstract] OR "follow up"[Title/Abstract])
